# Supplementary material for: Deconvolution of multiplexed transcriptional responses to wood smoke particles defines rapid aryl hydrocarbon receptor signaling dynamics
Source: J Biol Chem. 2021 Sep 11;297(4):101147. doi: 10.1016/j.jbc.2021.101147 (PMC8517214; doi:10.1016/j.jbc.2021.101147)
Supplement: Supporting File S1 [file mmc14.zip › JBC_101147_Supporting File S1/PRO-seq_Nextflow_pipeline_report/nascent_pipeline_report.html]

[angry\_mendel] Nextflow Workflow Report


Nextflow Report


- Summary
- Resources
- Tasks

[angry\_mendel]

# Nextflow workflow report

## `[angry_mendel]`

Workflow execution completed successfully!

Run times
:   Wed Jan 15 16:20:04 MST 2020 - Thu Jan 16 11:52:08 MST 2020
    (duration: **19h 32m 4s**)

94 succeeded

0 cached

0 ignored

0 failed

Nextflow command
:   ```
    nextflow run /Users/magr0763/Nascent-Flow/main.nf -profile hg38 --fastqs '/scratch/Shares/dowell/sasse/Woodsmoke_PRO_ATAC/PRO-seq/fastq/*fastq.gz' --workdir /scratch/Shares/dowell/sasse/Woodsmoke_PRO_ATAC/PRO-seq/temp --email margaret.gruca@colorado.edu --outdir /scratch/Shares/dowell/sasse/Woodsmoke_PRO_ATAC/PRO-seq --genome_id hg38 --singleEnd --forwardStranded --flip --nqc --counts --tfit --dastk --fstitch
    ```

CPU-Hours
:   `1'294.7`

Launch directory
:   `/scratch/Shares/dowell/sasse/Woodsmoke_PRO_ATAC/PRO-seq`

Work directory
:   `/scratch/Shares/dowell/sasse/Woodsmoke_PRO_ATAC/PRO-seq/temp`

Project directory
:   `/Users/magr0763/Nascent-Flow`

Script name
:   `main.nf`

Script ID
:   `a4d2e0a077e713b40403830d0e5b17a0`

Workflow session
:   `0e0340ea-5542-4676-8fde-8646716d661f`

Workflow profile
:   hg38

Workflow container
:   `skptic/nascentflow:latest`

Container engine
:   `-`

Nextflow version
:   version 19.04.1, build 5072 (03-05-2019 12:29 UTC)

## Resource Usage

These plots give an overview of the distribution of resource usage for each process.

#### CPU

- Raw Usage
- % Allocated

#### Memory

- Physical (RAM)
- Virtual (RAM + Disk swap)
- % RAM Allocated

#### Job Duration

- Raw Usage
- % Allocated

#### I/O

- Read
- Write

## Tasks

This table shows information about each task in the workflow. Use the search box on the right
to filter rows for specific values. Clicking headers will sort the table by that value and
scrolling side to side will reveal more columns.

Values shown as:

Human readable
Raw values

(tasks table omitted because the dataset is too big)

Generated by Nextflow, version 19.04.1
