# Supplementary material for: Deconvolution of multiplexed transcriptional responses to wood smoke particles defines rapid aryl hydrocarbon receptor signaling dynamics
Source: J Biol Chem. 2021 Sep 11;297(4):101147. doi: 10.1016/j.jbc.2021.101147 (PMC8517214; doi:10.1016/j.jbc.2021.101147)
Supplement: Supporting File S1 [file mmc14.zip › JBC_101147_Supporting File S1/PRO-seq_Nextflow_pipeline_report/pipeline_report.html]

NascentFlow Pipeline Report


# NascentFlow v1.3

## Run Name: angry\_mendel

NascentFlow execution completed successfully!

The workflow was completed at **Thu Jan 16 11:52:08 MST 2020** (duration: **19h 32m 4s**)

The command used to launch the workflow was as follows:

```
nextflow run /Users/magr0763/Nascent-Flow/main.nf -profile hg38 --fastqs '/scratch/Shares/dowell/sasse/Woodsmoke_PRO_ATAC/PRO-seq/fastq/*fastq.gz' --workdir /scratch/Shares/dowell/sasse/Woodsmoke_PRO_ATAC/PRO-seq/temp --email margaret.gruca@colorado.edu --outdir /scratch/Shares/dowell/sasse/Woodsmoke_PRO_ATAC/PRO-seq --genome_id hg38 --singleEnd --forwardStranded --flip --nqc --counts --tfit --dastk --fstitch
```

### Pipeline Configuration:

|  |  |
| --- | --- |
| Pipeline Name | ``` NascentFlow ``` |
| Help Message | ``` false ``` |
| Pipeline Version | ``` 1.3 ``` |
| Run Name | ``` angry_mendel ``` |
| Reads | ``` data/*_{1,2}.fastq.gz ``` |
| Fastqs | ``` /scratch/Shares/dowell/sasse/Woodsmoke_PRO_ATAC/PRO-seq/fastq/*fastq.gz ``` |
| Genome Ref | ``` /scratch/Shares/dowell/genomes/hg38/hg38.fa ``` |
| Thread fqdump | ``` NO ``` |
| Data Type | ``` Single-End ``` |
| Strandedness | ``` Forward ``` |
| Save All fastq | ``` NO ``` |
| Save BAM | ``` NO ``` |
| Save fastq | ``` NO ``` |
| Save Trimmed | ``` NO ``` |
| Reverse Comp | ``` YES ``` |
| Reverse Comp R2 | ``` NO ``` |
| Run Multicov | ``` YES ``` |
| Skip Trimming | ``` YES ``` |
| Nascent QC | ``` YES ``` |
| Run FastQC | ``` YES ``` |
| Run preseq | ``` YES ``` |
| Run pileup | ``` YES ``` |
| Run RSeQC | ``` YES ``` |
| Run MultiQC | ``` YES ``` |
| Skip All QC | ``` NO ``` |
| Max Memory | ``` 20 GB ``` |
| Max CPUs | ``` 1 ``` |
| Max Time | ``` 3d 4h ``` |
| Output dir | ``` /scratch/Shares/dowell/sasse/Woodsmoke_PRO_ATAC/PRO-seq ``` |
| FStitch | ``` YES ``` |
| Prelim Tfit | ``` NO ``` |
| Tfit | ``` YES ``` |
| DAStk | ``` YES ``` |
| dREG | ``` NO ``` |
| FStitch dir | ``` /scratch/Shares/dowell/FStitch/src/FStitch ``` |
| FStitch train | ``` /scratch/Shares/dowell/FStitch/train/hg38_train.bed ``` |
| Tfit dir | ``` /scratch/Shares/dowell/Tfit/src/Tfit ``` |
| Working dir | ``` /scratch/Shares/dowell/sasse/Woodsmoke_PRO_ATAC/PRO-seq/temp ``` |
| Container Engine | ``` null ``` |
| Current home | ``` /Users/magr0763 ``` |
| Current user | ``` magr0763 ``` |
| Current path | ``` /scratch/Shares/dowell/sasse/Woodsmoke_PRO_ATAC/PRO-seq ``` |
| Script dir | ``` /Users/magr0763/Nascent-Flow ``` |
| Config Profile | ``` hg38 ``` |
| E-mail Address | ``` margaret.gruca@colorado.edu ``` |
| Date Started | ``` Wed Jan 15 16:20:04 MST 2020 ``` |
| Date Completed | ``` Thu Jan 16 11:52:08 MST 2020 ``` |
| Pipeline script file path | ``` /Users/magr0763/Nascent-Flow/main.nf ``` |
| Pipeline script hash ID | ``` a4d2e0a077e713b40403830d0e5b17a0 ``` |
| Nextflow Version | ``` 19.04.1 ``` |
| Nextflow Build | ``` 5072 ``` |
| Nextflow Compile Timestamp | ``` 03-05-2019 12:29 UTC ``` |

NascentFlow

https://biof-git.colorado.edu/dowelllab/GRO-seq-workflow

https://github.com/Dowell-Lab/Nascent-Flow
